# On Certain Difficulties of Diagnosis

**Published:** 1898-03

**Authors:** J. P. Parkinson

**Affiliations:** Medical Registrar, Westminister Hospital, London, Eng.


ARTICLE X.

### ON CERTAIN DIFFICULTIES OF DIAGNOSIS.

BY J. P. PARKINSON, M. D., M. R. C. P.,

Medical Registrar, Westminister Hospital, London, Eng.

It is a frequent custom with medical men who are pressed for time, or not too well skilled in the physical signs of disease, to place too much reliance on the patient's account of his disease, and too little on those indications which he can elicit by examination; as an example of this error into which one may fall, I may quote the following case:

A patient came to see me some months ago in a state of great agitation; he complained of a severe cough, and stated he had two or three times expectorated a small quantity of blood, while the day previous he was attacked by a sharp pricking pain in the chest on drawing the breath.

At first sight these symptoms seem to warrant a diagnosis of phthisis, but, on examination, I noted that the temperature was quite normal, the chest showed no physical signs of disease, and on examination of the throat some congestion and signs of granular pharyngitis were evident. On questioning, I discovered that the patient had previously suffered from gout, and, in the absence of other causes, I judged this to be at the bottom of his disease, causing pharyngitis, from whence the blood had come. and a gouty neuralgia, or myalgia, to which the pain in the chest was due. I prescribed the usual purgatives, alkalies and colchicum, which were quickly followed by a complete cure. This description of pain in the chest, called, often, pleurodynia, is distinguished from simple pleurisy by the absence of friction sound, and by the temperature being normal, from that which may occur at the beginning of acute pneumonia, by the absence of dulness, fine rales or tubular breathing, and by the temperature being normal.

Pleurodynia also has to be distinguished from the pain which sometimes precedes the appearance of herpes zoster; this can be done only by the exact localization of the latter pain to the cutaneous areas of an intercostal nerve, and to the fact that this pain is cutaneous, and not deep-seated, as is the pain of pleurodynia.

Again, pain may be referred to the extremity of certain intercostal nerves, which, due to the pressure or irritation of disease elsewhere—for example, caries of the dorsal vertebræ—may cause as its chief symptom, pain in the front of the chest, and there may be but little evidence of disease of the spine, still, by careful searching, one of the vertebral spines may be found to project unduly, or to be out of the general line or percussion, or the application of a hot sponge, may elicit tenderness not suspected before.

The pressure, too, of an aneurism of the descending aorta, or a tumor in the posterior mediastinum, may cause pain referred to the front of the chest; but a thorough examination may lead to the discovery of some dulness in the situation of the tumor, or one side of the chest may show deficiency of expansion owing to pressure on a bronchus, and these signs, though slight, may suffice to show that some deep-seated disease is at the bottom of the pain in the chest, which might otherwise be treated uselessly by local applications.

Again, the diagnosis of the early stages of phthisis may be of the greatest difficulty, and the early symptoms are frequently misleading. The patients often complain of symptoms of anemia, such as languor, shortness of breath on exertion, palpitation of the heart, etc., or of dyspetic symptoms, pain in the abdomen, and vomiting; or of hoarseness of voice, which may be due to a simple catarrhal laryngitis, which so often accompanies phthisis, or to actual tubercular disease of the larynx. Wandering chest pains may often also be an early symptom, or an attack of true pleurisy; this latter disease; in the abscence of other causes, such as injury, kidney disease, or rheumatism, etc., is very frequently of tubercular origin, and if it recurs, is almost certainly so.

Such symptoms, especially if combined with emaciation, should suggest a thorough examination of the chest, and the weight of the patient should be taken for future reference, as a steady decrease of weight shows sometimes the progress of disease when the physical signs are very obscure.

Examination of the chest may show that one side moves slightly less than the other, especially at the apex, or deficiency of expansion at both apices. The vocal fremitus may be excessive at one or other apex, showing that slight consolidation of the lung tissue is beginning; and it must be remembered that the right side of the chest normally moves slightly more than the left, and that on right vocal fermitus is often better felt than on the lefe side.

Both these signs may be absent, and then auscultation may reveal an irregular or jerkey, the so-called "cog-wheel" breathing, most marked in inspiration, though this type of breathing may also be heard in highly nervous, but otherwise healthy, patients.

Again, the breathing may have a bronchial character, that is, may be high-pitched than normal, with a high-pitched and prolonged expiratory sound; and this character, to be at all significant of consolidation of the lung, must be marked in expiration, as well as in inspiration. The breath-sound may be short and faint, showing that the entry of air to that part of the lung is deficient, or without any great change in the character of the breath-sounds, fine rales may be heard at the end of a deep inspiration; and before saying that these are absent, it should be seen that a deep breath be taken, as sometimes rales, unheard before, then become evident. These rales, to be definitely indicative of phthisis, should be unaltered by coughing.

The character of these rales is of importance, as those occurring in early phthisis are usually small-sounding and sharp in quality, often resembling chicks. Over a portion of a lung affected with phthisis, the vocal responance is generally louder (bronchophony), and the heart-sounds may be conducted with more than normal intensity.

Any of the above signs, if constant over the period of a week or two, will raise a strong presumption of active phthisis, especially if combined with some of the symptoms mentioned above, and a general loss of flesh and strength.

The evening temperature should always be taken, as well as that of the morning, as frequently fever is only present in the evening, while the temperature in the morning is normal.

It must be mentioned that bronchial breathing is normal in the interscapular region at the back of the chest, and is often heard at the right apex in front and behind in women, and also that the breath-sounds of children are usually harsher than those of adults.

Many cases of phthisis begin by an attack of pleurisy, and, therefore, in all cases of this disease, the apices of the lungs should be carefully examined for tubercular disease, both in front and behind, as sometimes in one and sometimes in the other position the signs are most evident. An apical pleurisy is almost certainly of tubercular origin.

In any case of doubt the sputum, however small its amount, should be examined for the tubercle bucillus. The presence of this micro-organism makes the diagnosis certain, but the fact that it is not discovered is of little significance, except the examination be several times repeated by an observer competent to decide.

The mode of staining the sputum and its examination may easily be learned by anyone accustomed to microscopic methods, and should be more generally used by medical men than it is at present. The value of this means of diagnosis may be illustrated by the case of a man who was attached to an African exploring party. On arrival in England he felt very unwell and went to a hospital. When admitted, he was found to have an intermittent type of fever, varying from 99 per cent. to 101 per cent. F. There appeared no marked disease in any organ, and the blood was tested for malarial germs without success. After some weeks it was noticed that he expectorated once or twice during the day, and on examination the tubercle bacillus was found in this. No obvious signs of disease had up to then been found in the lungs, but some weeks later these became evident, and the diagnosis of phthisis was confirmed.

In women, again, the early signs of phthisis are often confounded with those of simple anemia, as the patient may complain of symptoms which could well be the result of simple anemia. Hence, in every case of anemia the chest should be examined carefully, and any abnormal signs noted. It should also be insisted on, in the case especially of women and children, that a deep breath be taken during auscultation, as often a slight moist sound may then be heard at the end of inspiration, which otherwise would not be elicited. The diagnosis of phthisis in a patient who has much emphysema, may be very difficult by physical signs alone, as the resonance of the expanded lung may mask both any dulness that consolidation might give rise to, and also faint rales may be obscured by the emphysema, or by the bronchitis which so frequently accompanies this condition.

Again, if the signs of phthisis have been definitely made out, it does not follow that this is the whole of the morbid condition. In this, as in all other cases of disease, the urine should be tested for albumin or sugar, as the phthisis may be only a part of diabetes, or the kidneys may be affected by some tubercular process, or by the lung disease, so as to cause a very different prognosis to that which would be given if the urine were found to be normal.

The determination whether sugur be or be not present in the urine should always be done in one particular way: The proper amount of copper sulphate should be kept in solution in one bottle, and the caustic potash and potassium sodium tartrate in another, and only when required should then be mixed in a test tube. This should then be heated to boiling, and if any precipitate occur freshly prepared solutions should be used; then, when boiling, one or two drops of the urine should be added, and if much sugar be present, a copious yellow precipitate forms. If no precipitate occurs, the urine should be added equal in amount to that of the test solution, when on boiling, if sugar be present, the precipitate mentioned above will appear. There are certain fallacies, however, to be guarded against when this test is employed. First, frequently a yellowish cloud may appear when much urine is added to the alkaline test solution, which merely consists of phosphate precipitated owing to the effect of the alkali on the urine, but this cloud has not the bright yellow color of the true sugar precipitate.

Secondly, if much uric acid, hippuric acid or ceratinine be present, a very slight reduction may occur, even if there be no sugar in the urine.

Thirdly, after the administration of morphine, chloroform, chloral hydrate, butychloral, or camphor, a substance called glycuronic acid is found in the urine, and this gives exactly the same reaction with the copper solution as sugar. That the reduction is due to this cause is seen by the fact that glycuronic acid does not ferment with yeast as does sugar, and hence the fermentation test will distinguish between them.

This fermentation test can most simply be applied by filling completely a test tube with urine, and adding a small fragment of yeast, and inverting the test tube over a saucer filled with urine; then, after standing for some hours in a warm place, if sugar be present in the urine, it will be seen that an evolution of gas from the urine has displaced the upper part of that fluid in the test tube, and if the specific gravity of the urine be taken, it will be found to be less than before, owing to the fermentation of the sugar forming alcohol and carbonic acid gas.

The discovery of much sugar in the urine of a patient whose lungs show signs of phthisis, should suggest that the lung condition is secondary to the diabetes, and in this case the prognosis is much graver than if the lung disease be of primary tubercular origin.

In the examination of the urine for albumin, there are many possible fallacies to be avoided.

Two tests are most frequently employed, the cold nitric acid test, and the heating and acidulation test. The cold nitric acid test is best used by pouring about half an inch deep of nitric acid into a test tube, and then gently pouring in urine while the test tube is held in a slanting position; in this way the urine forms a layer separate from the nitric acid; if albumin be present, either at once, or in a minute or two, there will appear in the urine, just above the junction of the liquids, a white deposit of coagulated albumin. The fallacies of this test are the following: If the urine be concentrated, and contain an excess of urates, these will be precipitated as a cloud, which, however, appears at some distance above the nitric acid, and the application of a gentle heat will at once dissolve them, which will not happen to any precipitated albumin; again, the urine of persons taking copabia will, when treated as above, show a general cloud diffused evenly through it; this precipitate again is dissolved by heat; finally, in many urines, the addition of nitric acid gives rise, after standing for some time, to the deposit of lamellar crystals, which consist of nitrate of urea.

The test for albumin in the urine by heat is best done by half filling a test tube with urine, and, while holding it by the base, heating the upper part, when, if albumin be present, a cloud, or precipitate, will appear in the heated portion.

If the urine be turbid, and this turbidity does not disappear on heating, it must be filtered before testing for albumin.

Heat will frequently precipitate earthy phosphates, which form a cloud not to be distinguished from albumin by the eye, but this precipitate will at once dissolve on the addition of acetic or nitric acid, while that due to albumin will remain unaltered

Again, albumin may be present in urine and yet not be precipitated by heat. This occurs if the urine is alkaline, when, on heating, alkali albumin is formed, which is not coagulated by heat. To guard against this fallacy, if the urine be alkaline, it is necessary to add a drop or two of acetic acid previously to boiling, so as to render it weakly acid; but if the urine be too highly acid, albumin, if present in small quantity, may be converted, into acid albumin, which, also, is not precipitated by heat, hence the caution to add only a very small amount of acetic acid.

In any case, a clear urine is necessary for the heat test; if the urine be cloudy from urates, a gentle heat dissolves them; if from phosphates, the addition of acetic acid will make it clear, but if any other substance render it cloudy, the urine should be filtered.

The presence of albumin in the urine of a phthisical patient may be due to the following causes:

1. Simple congestion, in cases where the heart's action is hampered, and the abdominal organs are generally congested.

2. Lardaceous, or amyloid disease, which is a part of a general cachexia, occurring in cases of long-continued phthisis.

3. Tubercular disease of the kidney.

4. Acute or chronic nephritis, combined with phthisis.

In all the above conditions, naturally, the kidney disease increases the gravity of the case, and makes the prognosis worse than if no albuminuria was present.

The diagnosis of fluid within the chest is a point which frequently gives rise to much difficulty. The final decision may have to depend on an exploratory puncture, but before this is done it is as well to have a fairly reasonable idea that fluid is present.

The important points are, firstly, the complete abolition of vocal fremitus. If the patient be a man with a good voice, there is no other condition under which this fremitus is absent, except occlusion of the bronchus, or pneumothorax. It may, however, be diminished when the pleura is simply thickened by fibrous tissue, but often when women or children are the patients, vocal fremitus can not be obtained over any part of the chest.

The second important physical sign of fluid is absolute dulness to percussion, with a feeling of much increased resistance to the percussing finger. There is no other condition which gives such intense dulness as fluid in the chest.

The third important sign of fluid, if it exist in any quantity, is the displacement of the heart to the opposite side of the chest. If the fluid be on the right side of the chest, the heart's apex is displaced outside the nipple line, and may even be seen and felt to beat in the left axilla; while, if the fluid be on the left side of the chest, the heart is displaced to the right, and the heart's dulness extends beyond the right edge of the sternum. In this case the apex beat is often not visib'e or palpable, as it may be hidden by the sternum, but the alteration of the heart's dulness, and the fact that the heart sounds are heard more to the right than usual, are sufficient for diagnosis.

The above are the physical signs upon which most reliance may be placed for a diagnosis of fluid within the chest. It is true that a thickened pleura, due to old pleurisy, will give diminution of vocal fremitus and more or less dulness, but the heart if displaced at all, will be pulled toward the side of the disease, and not pushed away from it; and the chest, instead of being more or less bulged on the diseased side, will be seen to be retracted. The breath sounds over a pleural effusion are usually weak, or altogether lost, but sometimes, especially if the stethoscope be placed near the spinal column, the breat sounds may be loud and bronchial, or even tubular. This has sometimes led to an incorrect diagnosis, not sufficient weight having been placed on the physical signs before mentioned.

The peculiar nasal or bleating sound, called ægophony —which is often heard about the angle of the scapula when pleural effusion is present—is often much relied on for the diagnosis of the presence of fluid in the pleura. It is undoubtedly very frequently present, but its absence does not at all militate against fluid being present. It is now generally the rule, except very much fever be present, when fluid is suspected, to puncture the chest with an exploring syringe, and to draw off a specimen of the fluid, and this plan is much to be commended. Certain precautions must, however, be taken. The syringe must be inserted into a part of the chest which is undoubtedly dull on percussion, and the syringe must be in good condition and the needle patent. These observations may seem to be unnecessary, but I have seen cases where it was said fluid was not present, owing to its having been overlooked. Then the needle must be inserted sufficiently deeply, as sometimes a flake of lymph or a mass of phoid tissue may be thick enough to prevent the needle reaching the fluid, or occasionally a needle previously carbolized coagulates the serum in its lumen, and so prevents the fluid entering the syringe. Again, if the fluid be pus a needle with a sufficiently large lumen must be employed, otherwise thick pus will not pass through.

The determining whether fluid in the chest be serous, or purulent, is difficult, often impossible, before aspiration; the chief points to be relied on are the following: The long continuance of an effusion suggests that it may be purulent, though in some cases fluid know to have been in the chest for many months has proved serious. If in a case of effusion which has lasted some weeks, the temperature be raised, it is probable the fluid is purulent, but the converse is not the case, as often pints of pus are removed from a pleura of a patient whose temperature has not been raised above the normal limit. Fluid which is effused in the course of an acute pneumonia, or some of the acute specifics, notably typhoid fever, is often purulent. Again, fluid in the pleura complicating Bright's disease, or other morbid blood states, is generally purulent, as often is the fluid arising during the course of phthisis.

Edema of the chest wall over the fluid is strongly in favor of empyema, and so is the fact that the fluid is pointing under the skin from between the ribs. Empyema is said to transmit the voice sounds better than serous pleurisy, but this can not be relied upon. Perhaps the best indications that the fluid is purulent is when the patient becomes sallow and anemic, and the finger-ends become clubbed, but these symptoms only arise when the fluid has existed some time, and an earlier diagnosis is much to be desired; hence, in any doubtful case the introduction of an exploring needle of sufficient size is of the greatest value.

This is especially the case in those small empyemata which are so frequent, and so difficult of detection in young children, as the only physical sign they often give is local dulness, while the child, if untreated, goes from bad to worse, and may finally die if nothing curative be attempted.

The bringing up of blood by the mouth is very frequently difficult to ascertain the cause of; it may come trom the lungs, or stomach, or from the nose, mouth, fauces, larynx, etc. The characteristics of blood from the lungs and stomach are given in tabular form from my work on Clinical Diagnosis.:*

| *Hemoptysis.* | *Hematemesis.* |
|---|---|
| Generally a history of cough. | Often previous nausea or indigestion. |
| Blood is coughed up. | Blood is vomited. |
| Blood is bright, and often frothy. | Blood often dark or clotted, and not usually frothy. |
| Blood may be mixed with sputum. | Blood may be mixed with food. |
| Blood always alkaline. | Blood generally acid. |
| Sputum is streaked with blood the nex day. | No after-expectoration of blood. |
| No blood passed per rectum, *except* some *has* been swallowed. | May be followed by melena. |

There are, however, a large number of cases in which blood, generally in small amount, is brought up, which does not come under the above heads; for example a patient may find a slight blood stain on the pillow in the morning; this frequently is due to a slight bleeding of the gums, which is not uncommon in anemic patients, and has no serious significance; an inspection of the gums may show a slight abrasion, or bleeding point, or a speck of coagulated blood. If expectorated, this blood from the mouth is usually thin and diluted with saliva.

Hemoptysis in children is most frequently due to

*Clinical Diagnosis, by J. P. Parkinson, M. D., Balliere, Tindall & Cox, London, Eng.

adenoid vegetations in the pharynx, which cause also nasal disease, snoring, mouth breathing, and a tendency to pharyngeal and nasal catarrh; these growths are easily felt by passing the guarded finger behind the soft palate, when a soft, jelly-like mass will be felt, and the finger, when withdrawn, will be found to be generally blood-stained.

Hemoptysis is sometimes due to bleeding from the back of the nose in cases of inflammation of its mucous membrane; in this case the blood is usually dark and coagulated, and in pellets often mixed with dried nasal secretion.

Blood may also come from the pharynx, and in no case should it be decided that blood comes from the lungs without an examination of the pharynx, in which there may be discovered the point from which the blood comes. Blood is often expectorated, due to the bursting of a pharyngeal blood vessel in a patient suffering from pharyngitis.

Finally, blood may come from the larynx, and the diagnosis of this is, perhaps, the most difficult of all, as the blood may have all the characters of blood from the lungs; however, some hoarseness, or other laryngeal symptom may draw attention to that organ, and a laryngoscopic examination may show an abrasion or ulcer which has caused the symptom.

Cough, again, is a symptom which is often wrongly put down to chest disease; this error is specially frequent in children, as in them about seventy-five per cent of the coughs are due to either adenoid vegetations, enlarged tonsils, or pharyngitis; in these cases the cough is, as a rule, worse at night, and on auscultation of the chest, as a rule, a few bronchi will be heard; but these moist sounds are not the cause of the cough, and treatment by carbonate of ammonia and ipecacuanna will certainly fail, while treatment directed to the local throat condition will as certainly cure. Elongation of the uvula also is a frequent cause of night cough, which will certainly be overlooked without the throat be examined.

Other less frequent causes of cough, are gastric irritation, the presence of wax or other foreign body in the external auditory meatus, and occasionally, it is said, cough may be due to dental causes, or other cause of irritation of the dental nerves.

Enough has been said to show how important it is not to take symptoms on their own merits alone, but to qualify them by a thorough examinination of the patient, for it is very common for a patient to unconsciously exaggerate any symptoms he experiences, and if leading questions be put by the medical man, to reply in the manner which may be suggested by the doctor. Questions must, of course, be asked, but they should be put, if possible, in a form which does not suggest any simple affirmation or denial on the part of the patient, but rather should attempt to elicit the patient's own more complete description of his troubles.—*Medical Brief.*

